# Potential for Tick-borne Bartonelloses

**DOI:** 10.3201/eid1603.091685

**Published:** 2010-03

**Authors:** Emmanouil Angelakis, Sarah A. Billeter, Edward B. Breitschwerdt, Bruno B. Chomel, Didier Raoult

**Affiliations:** Université de la Méditerranée, Marseille, France (E. Angelakis, D. Raoult); North Carolina State University College of Veterinary Medicine, Raleigh, North Carolina, USA (S.A. Billeter, E.B. Breitschwerdt); University of California School of Veterinary Medicine, Davis, California, USA (B.B. Chomel)

**Keywords:** *Bartonella* species, ticks, PCR, arthropod vector, transmission, bacteria, parasites, perspective

## Abstract

As worldwide vectors of human infectious diseases, ticks are considered to be second only to mosquitoes. Each tick species has preferred environmental conditions and biotopes that determine its geographic distribution, the pathogens it vectors, and the areas that pose risk for tick-borne diseases. Researchers have identified an increasing number of bacterial pathogens that are transmitted by ticks, including *Anaplasma*, *Borrelia, Ehrlichia*, and *Rickettsia* spp. Recent reports involving humans and canines suggest that ticks should be considered as potential vectors of *Bartonella* spp. To strengthen this suggestion, numerous molecular surveys to detect *Bartonella* DNA in ticks have been conducted. However, there is little evidence that *Bartonella* spp. can replicate within ticks and no definitive evidence of transmission by a tick to a vertebrate host.

## Abstract 2

Although possible, tick transmission to a vertebrate host has not been proven.

*Bartonella* spp. are gram-negative bacilli or coccobacilli that belong to the α-2 subgroup of *Proteobacteria.* According to 16S rDNA gene comparisons, they are closely related to the genera *Brucella* and *Agrobacterium* (*1*). A remarkable feature of the genus *Bartonella* is the ability of a single species to cause either acute or chronic infection that can cause either vascular proliferative lesions or suppurative and granulomatous inflammation. The pathologic response to infection with *Bartonella* spp. varies substantially with the status of the host’s immune system; vasoproliferative lesions are most frequently reported for immunocompromised patients. To date, 13 *Bartonella* species and subspecies have been associated with an increasing spectrum of clinical syndromes in humans, including cat-scratch disease and chronic bacteremia (*B. henselae*), bacillary angiomatosis (*B. henselae*, *B. quintana*), peliosis hepatitis (*B. henselae*), bacteremia and/or endocarditis (*B. henselae*, *B. quintana, B. elizabethae*, *B. vinsonii* subsp. *arupensis*, *B. vinsonii* subsp. *berkhoffii*, *B. koehlerae*, and *B. alsatica*), Carrión disease (*B. bacilliformis*), trench fever (*B. quintana*), retinitis and uveitis (*B. henselae*, *B. grahamii*), myocarditis (*B. vinsonii* subsp. *berkhoffii*, *B. washoensis*), splenomegaly (*B. bacilliformis, B. henselae, B. rochalimae*), and fever and fatigue (*B. henselae, B. vinsonii* subsp. *berkhoffii*, *B. tamiae*) (*1**–**3*).

## Ticks

Ticks were first identified as potential vectors of *Babesia bigemina,* the agent of Texas cattle fever, in 1893 (*4*). There are 2 major tick families (≈865 tick species worldwide): the Ixodidae, or hard ticks, characterized by a sclerotized dorsal plate, and the Argasidae, or soft ticks, characterized by their flexible cuticle. A third family, the Nuttalliellidae, is represented by a single species that is confined to southern Africa. The genus *Ixodes*, family Ixodidae, contains >200 species, of which 14 make up the *I. ricinus* complex (*4*). Among these 14 species, *I. scapularis*, *I. pacificus*, *I. ricinus*, and *I. persulcatus* ticks are involved in the transmission of the *Borrelia burgdorferi* complex, which is a prevalent cause of Lyme disease in persons in the Northern Hemisphere.

Ticks in various regions of the world are vectors for bacterial, viral, and protozoal pathogens (*5*). Ticks may act not only as vectors but also as reservoirs of tick-transmitted bacteria that are transmitted transstadially and transovarially in a tick species (e.g., certain *Rickettsia* spp. and *Borrelia* spp.) (*5*). When feeding on an infected small-mammal host, larvae and nymphs can ingest >1 pathogens while obtaining a blood meal. Some organisms are then passaged to the next stage in the tick life cycle and can be transmissible during the subsequent blood meal (*5*). For each tick species, the optimal environmental conditions determine the geographic distribution; the spectrum of tick-borne pathogens; and as a result, the geographic areas of risk for tick-borne diseases, particularly when ticks are both vectors and reservoirs of specific pathogens.

Hard ticks are the primary vectors of a variety of bacterial pathogens, including *Anaplasma* spp., *Borrelia* spp., *Ehrlichia* spp., *Coxiella burnetii*, and *Rickettsia* spp (*5**–**7*). *Anaplasma phagocytophilum* is transmitted by *I. persulcatus*–complex ticks, including *I. scapularis*, *I. pacificus,* and *I. ricinus*, whereas *Ehrlichia chaffeensis* and *Ehrlichia ewingii* are transmitted by *Amblyomma americanum* ticks (*5**,**6*). Although some pathogens are carried by a single or limited number of tick species, other organisms such as *Coxiella burnetii* have been identified in >40 tick species (*7*). Lyme disease, caused by *B. burgdorferi*, is transmitted by *I. scapularis* and *I. pacificus* ticks within the United States, by *I. ricinus* ticks in Europe, and by other *Ixodes* spp. ticks in the Northern Hemisphere (*5**,**8*). Although specific *Bartonella* spp. are transmitted by blood-sucking arthropods, including fleas, lice, or sandflies, the only evidence to support the possibility of tick-borne transmission is indirect.

We present an overview of the various *Bartonella* spp. that have been detected in ticks and discuss human cases of *Bartonella* infection that are suggestive of tick transmission. Because of the rapidly expanding number of reservoir host–adapted *Bartonella* spp. that have been discovered in recent years, efforts to clarify modes of transmission are relevant to public health in terms of interrupting the transmission process. As evolving evidence supports the ability of this genus to induce chronic intravascular infections in humans, improved understanding of vector competence could facilitate efforts to block pathogen transmission, which would help improve human health (*9*).

## Host Associations and Specificity

*Bartonella* spp. have a natural cycle of chronic intravascular infection in a reservoir host and a sustained pattern of bacterial transmission by a defined and evolutionarily well-adapted vector from the reservoir hosts to new susceptible hosts. Current information leads to the presumption of a long-standing and highly adapted species-specific association between a given *Bartonella* sp. and the preferred animal host and vector (*10*). Inadvertent infection of persons with at least 13 *Bartonella* spp. has resulted in a wide spectrum of disease manifestations. After primary infection of the natural mammalian host, a chronic, relapsing, nonclinical bacteremia occurs. At times, in wild and stray animal populations, including cats, cows, and various rodent species, the prevalence of infection within the population can approach 100% (*1*). Although the geographic distribution of a specific *Bartonella* sp. may reflect the geographic distribution of its hosts or vectors, knowledge related to vector transmission of *Bartonella* organisms remains inadequate.

### *Bartonella* spp. DNA in Ticks

As an initial effort to define tick species that might serve as competent vectors for transmission of *Bartonella* spp., molecular epidemiology surveys to identify *Bartonella* spp. DNA in ticks have been conducted (*2*). *Bartonella* spp. have mostly been identified by PCR using primers targeting either specific *Bartonella* genes like the citrate synthase gene (*glt*A) gene, the riboflavin synthase gene, the heat shock protein gene (*gro*EL), the 16S–23S intergenic spacer, the heme binding protein gene, and the cell division protein gene or the 16S rDNA gene (Table 1). Summarized results indicate that the proportion of ticks harboring *Bartonella* DNA can vary from low prevalences of 0.43% among questing *A. americanum* ticks examined in the southeastern United States (*3*) and 1.2% of *I. ricinus* ticks collected in the Czech Republic (*24*) to a prevalence as high as 60% in *I. ricinus* ticks from roe deer in the Netherlands (*20*) (Table 1). *Bartonella* spp. from various locations tend to differ. For example, *Bartonella* DNA related to *B. doshiae, B. rattimassiliensis*, and *B. tribocorum* has been identified in ticks only in Asia, *B. bacilliformis*–like DNA and *B. capreoli* in ticks only in Europe, and *B. washoensis, B. tamiae*–like DNA, and *B. vinsonii* subsp. *berkhoffii* in ticks only in the United States (Figure).

**Table 1:** Ticks in which *Bartonella* spp. DNA has been found*

Tick genus and species	Prevalence of *Bartonella* spp. DNA in ticks, %/no.	Identified *Bartonella* spp.	Target gene	Reference
*Amblyomma americanum*	0.43/466 individuals	*B. tamiae*–like	IGS	(*3*)
*Carios kelleyi*	3.2/31 individuals	Resembling *B. henselae*	IGS	(*11*)
*Dermacentor occidentalis*	8.3/12 pools	*Bartonella* spp.	*glt*A	(*12*)
*D. reticulatus*	21.4/84 individuals	*B. henselae* (99% homology) and *B. quintana* (90% homology)	*gro*EL	(*13*)
*D. variabilis*	14.3/ 7 pools	*Bartonella* spp.	*glt*A	(*12*)
*Haemaphysalis flava*	2.7/74 pools	*Bartonella* spp.	16S rRNA	(*14*)
*H. longicornis*	4.4/1,173 pools	*Bartonella* spp.; 1 pool harbored *B. rattimassiliensis* (99.2%), 1 pool harbored *B. tribocorum* (98.3%)	16S rRNA	(*14*)
*H. longicornis*	36/150 groups (60 individual fed adults, 30 pools of 2 unfed adults, and 60 pools of 5 nymphs)	*Bartonella* spp.	*glt*A	(*15*)
*Ixodes nipponensis*	5.0/20 pools	*Bartonella* spp.	16S rRNA	(*14*)
*I. pacificus*	19.2 of 151 individuals	*B. henselae, B. quintana, B. washoensis, B. vinsonii subsp. berkhoffii*, and a *Bartonella* cattle strain	*glt*A	(*16*)
*I. pacificus*	11.6/224 pools	*Bartonella* spp.	*glt*A	(*12*)
*I. persulcatus*	37.6/125 individuals	*B. henselae* (99% homology) and *B. quintana* (90% homology)	*gro*EL	(*13*)
*I. persulcatus*	44/50 individuals in 2002 and 38/50 individuals in 2003	*B. henselae*	*gro*EL	(*17*)
*I. persulcatus*	33.3/3 pools	*Bartonella* spp.	16S rRNA	(*14*)
*I. ricinus*	1.48/271 individuals	*B. henselae*	*gro*EL, *pap*31, *fts*Z	(*18*)
*I. ricinus*	4.9/102 individuals	*B. henselae*	*glt*A	(*19*)
*I. ricinus*	60/121 individuals	*Bartonella* spp	16S rRNA	(*20*)
*I. ricinus*	A pool/12 ticks	*Bartonella* spp	16S rDNA	(*21*)
*I. ricinus*	9.8/92 individuals	*Bartonella* spp.; 1 adult harbored *B. schoenbuchensis* (96% homology)	*gltA*	(*22*)
*I. ricinus*	7.7/103 individuals	*B. capreoli*	ITS	(*23*)
*I. ricinus*	1.2/327 individuals	*Bartonella* spp	16S rRNA	(*24*)
*I. ricinus*		Resembling *B. bacilliformis*†		(*25*)†
*I. scapularis*	2.0/203 individuals	*B. schoenbuchensis*	*gltA*	(*26*)
*I. scapularis*	34.5/107 individuals	Unidentified *Bartonella* spp.	16S rRNA	(*27*)
*I. scapularis*		*B. henselae*	16S rRNA	(*28*)
*I. sinensis*	16.3/86 individuals	*Bartonella* spp.	*glt*A	(*15*)
*I.* spp.	42.3/26 pools	*Bartonella* spp.	16S rRNA	(*17*)
*I. turdus*	11.1/9 pools	*Bartonella* spp.; 1 pool harbored *B. doshiae* (99.2% homology)	16S rRNA	(*14*)
*Rhipicephalus sanguineus*	3.2/62 individuals	*B. henselae*	*rib*C	(*29*)
Unidentified tick species		*Bartonella* sp.	IGS	(*30*)

*IGS, intergenic spacer; *glt*A , citrate synthase gene; *gro*EL, heat-shock protein gene; *pap*31, heme-binding protein gene; *fts*Z , cell-division protein gene; *rib*C, riboflavin synthase gene. †*Bartonella* spp. ascertained by isolation.

**Figure:**
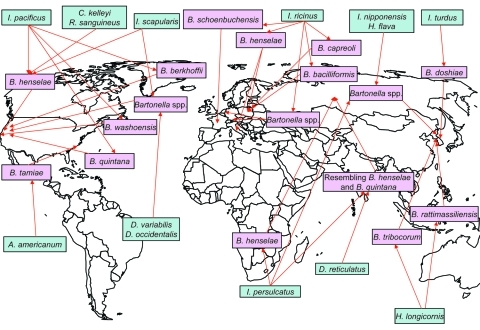
Worldwide locations of ticks (blue boxes) identified with *Bartonella* spp. (pink boxes). *I*., *ixodes; C*., *Carios*; *R*., *Rhipicephalus*; *B.*, *Bartonella*; *H.*, *Haemaphysalis*; *A*., *Amblyomma*; *D*., *Dermacentor.*

### Evidence for Co-infections in Ticks

In recent years, emphasis on the potential transmission of multiple pathogens by an individual tick after attachment to an animal or person has grown. While studying different tick populations throughout the world, several researchers have identified *Bartonella* DNA in conjunction with known tick-transmitted organisms. Adelson et al. tested for the prevalence of *B. burgdorferi*, *Babesia microti*, *A. phagocytophilum*, and *Bartonella* spp. in 107 *I. scapularis* ticks collected in New Jersey (*27*). A large percentage of ticks (45.8%) contained DNA from at least 1 of these organisms, and 34.5% of ticks screened harbored *Bartonella* spp. DNA. Of the ticks positive for *Bartonella* by PCR, 9 (8.4%) contained *B. burgdorferi* DNA, 1 (0.9%) contained *B. microti* DNA, 1 (0.9%) contained *A. phagocytophilum* DNA, 1 (0.9%) contained both *B. burgdorferi* and *A. phagocytophilum* DNA, and 1 (0.9%) contained *B. microti* and *A. phagocytophilum* DNA (*27*). Although the primers in this study were originally selected for the species-specific amplification of *B. henselae*, this region of the *Bartonella* 16S rDNA gene is highly conserved among many *Bartonella* spp. In a study performed in France, Halos et al. screened 92 questing *I. ricinus* ticks and determined that 9.8% contained *Bartonella* DNA by using *glt*A-specific primers (*22*). *Bartonella* *schoenbuchensis*–like DNA (96% homology) was detected in 1 of the adult ticks tested. The authors also reported that 1% of the ticks contained *Bartonella* spp. and *B. burgdorferi* DNA, 4% contained *Bartonella* and *Babesia* spp. DNA, and 1% contained *Bartonella* spp., *B. burgdorferi*, and *Babesia* spp. DNA (*22*). Of 168 questing adult *I. pacificus* ticks from Santa Cruz County, California, screened for *Bartonella* DNA, 11 (6.55%) contained *B. henselae* genotype I DNA (*31*). Of the *Bartonella*–positive ticks, 1.19% also harbored *B. burgdorferi* DNA and 2.98% harbored *A. phagocytophilum* DNA (*31*). Loftis et al. tested *Carios kelleyi* ticks, argasid tick species found on bats, from residential and community buildings in Iowa, for *Anaplasma, Bartonella*, *Borrelia*, *Coxiella*, and *Rickettsia* spp. One tick was found to contain *Bartonella* and *Rickettsia* DNA, and the DNA sequence was most closely related to *B. henselae* (*11*). Recently, Sun et al. examined *Haemaphysalis longicornis* and *I. sinensis* from People’s Republic of China for *Borrelia*, *Bartonella*, *Anaplasma*, and *Erhlichia* spp. (*15*). Of adult and nymphal *H. longicornis* ticks collected in the cities of Benxi and Liaoyang, 36% of 150 groups (60 individual host-associated adults, 30 pools of 2 questing adults, and 60 pools of 5 nymphs) harbored detectable *Bartonella* DNA. Furthermore, 16.3% of 86 individual *I. sinensis* ticks (all host-associated adults) from the cities of Tiantai, Jindong, and Jiangshan contained *Bartonella* DNA. One tick harbored all 4 bacteria (*Borrelia*, *Bartonella*, *Anaplasma*, and *Ehrlichia* spp. DNA), and a second tick pool was positive by PCR for *Borrelia*, *Bartonella*, and *Ehrlichia* spp (*15*).

### Evidence of Potential Tick *Bartonella* spp. Transmission to Humans

In 1992, *B. henselae* infection developed in 2 previously healthy, immunocompetent men within weeks of a tick bite (*32*) (Table 2). Both patients reported signs and symptoms generally associated with *B. henselae* infection: fever, muscle and joint pain, headache, and photophobia. The first patient did not recall being bitten or scratched by a cat, the general mode of *B. henselae* transmission to humans. *B. henselae* organisms were cultured from the blood of both patients and confirmed by PCR. To our knowledge, this was the first case report to suggest that ticks may be responsible for transmission of *Bartonella* spp. in humans. More recently, *B. henselae* was isolated from a boy who had severe intractable migraine headaches 10 days after an attached tick was removed from his leg, although on the basis of seroconversion, infection with *B. vinsonii* subsp. *berkhoffii* was suspected (*9*). Breitschwerdt et al. concluded that the boy was either co-infected or chronically infected with *B*. *henselae*, the organism isolated, and subsequently infected with *B. vinsonii* subsp. *berkhoffii*, as reflected by the documentation of seroconversion.

**Table 2:** Evidence of *Bartonella* spp. infection in persons after tick bite

Agent	Tick species	Tick bite	Animal contact	Clinical manifestation	Year	Reference
*B. henselae*	Unknown	Yes	No cat	Fever, myalgia, arthralgia, headaches, and light sensitivity	1992	(*32*)
*B. henselae*	Unknown	Yes	Cat	Fever, myalgia, arthralgia, headaches, and light sensitivity	1992	(*32*)
*B. henselae*	Unknown	Yes	Cats and kitten	Cat-scratch disease signs	1993	(*33*)
*B. henselae*, *Borrelia burgdorferi*	Possibly *Ixodes scapularis*	Yes	Not mentioned	Low-grade fever, headaches, fatigue, knee arthralgia, and insomnia	2001	(*28*)
*B. henselae, B. burgdorferi*	Possibly *I. scapularis*	Yes	Not mentioned	Fever, headache, dizziness, fatigue, and arthralgia	2001	(*28*)
*B. henselae, B. burgdorferi*	Unknown	Not mentioned	Not mentioned	Meningitis	2003	(*34*)
*B. henselae* or *B. quintana* seroreactive	Unknown	Yes	Not mentioned	Fever	2003	(3*5*)
*B. burgdorferi*, *B. henselae*, *B. quintana*	Unknown	Yes	Not mentioned	Fever	2003	(3*5*)
*Bartonella* spp. closely related to *B. henselae*, *B. quintana*	Unknown	Yes			2005	(*36*)
*B. henselae* and/or *B. vinsonii* subsp. berkhoffii*	Unknown	Yes	Cats, dogs, potentially other animal species	Fatigue, insomnia, arthralgia, myalgia, headache, and/or tremors	2007	(*37*)
*B. henselae*, and/or *B. vinsonii* subsp. *berkhoffii*†	Unknown	Yes	Cats, dogs, other animal species	Seizures, ataxia, memory loss, tremors, fatigue, and/or headaches	2008	(*9*)

*Patients were also seroreactive to *B. henselae* and/or *B. vinsonii* subsp. *berkhoffii.* †Patients were also seroreactive to *B. henselae*, *B. vinsonii* subsp. *berkhoffii,* and/or *B. quintana*.

In a clinical study, Zangwill et al. were interested in identifying risk factors associated with development of cat-scratch disease (*33*). The epidemiologic survey, performed in Connecticut, contained 56 cat-scratch disease patients and their controls (persons who owned or had been in contact with cats). They used a modified random-digit dialing technique to recruit controls, and they identified 60 patients with cat-scratch disease. However, of the 60 patients whose illnesses met the case definition, 4 were not successfully matched with controls for age and cat ownership; therefore, 56 patients and their controls were enrolled in the case–control study. The controls did not differ significantly from the patients by race, sex, family size, level of maternal education, or socioeconomic status. Answers to questionnaires suggested that cat-scratch disease was more likely to occur in patients than in controls if the person owned a kitten, had contact with a kitten with fleas, or had been bitten or scratched by a kitten. Of the 56 patients, 21% were also more likely than controls to have been bitten by a tick, although bivariate analysis did not demonstrate a significant association between tick bite and cat-scratch disease development (*33*).

Other case reports have suggested potential human co-infections with *Bartonella* spp. and a known tick-transmitted organism. Eskow et al. described 4 cases in which patients from central New Jersey reported several neurologic symptoms, including headache, fatigue, insomnia, and depression, which may have resulted from Lyme disease (caused by *B. burgdorferi*) (*28*). However, other causes for their cognitive dysfunctions cannot be ruled out. Of these 4 patients, 2 had histories of Lyme disease, and 3 had *B. burgdorferi* DNA in the cerebrospinal fluid (CSF). One patient exhibited no laboratory evidence of Lyme disease, suggesting that these symptoms might have been caused by an agent other than *B. burgdorferi*. However, 2 patients reported illness within 1 week to 3 months after being bitten by a tick. Upon further investigation, all patients were seroreactive to *B. henselae*; immunofluorescence assay showed immunoglobulin (Ig) G titers of 64–256. According to the authors, *B. henselae* DNA was amplified from blood of 1 patient, from CSF of 1 patient, and from both blood and CSF of the other 2 patients (*B. burgdorferi* DNA also was detected in the CSF of these 2 patients). Ticks, identified as *I. scapularis*, found in 2 patients’ homes potentially harbored both *B. henselae* and *B. burgdorferi* DNA. Whether *B. henselae* was specifically detected in this case series is unclear because sequencing of amplicons was not performed and because the PCR primer set targeted the *Bartonella* 16S rRNA, a highly conserved region. Without sequencing of amplicons or confirmation of results by targeting a more highly variable gene, ascertaining whether *B. henselae* was present in the ticks or in the patients would be difficult. However, the results derived from these cases are of interest because, to our knowledge, this was the first case series to propose simultaneous detection of both *B. burgdorferi* and *Bartonella* DNA in the CSF of patients with neurologic signs.

In another study, 2 of 17 patients from Poland with symptoms suggestive of neuroborreliosis seemed to be co-infected with *B. burgdorferi* and *B. henselae* (*34*). *B. burgdorferi*–specific antibodies were detected in a patient whose CSF also had detectable *B. henselae* DNA. The other patient was seroreactive to both *B. burgdorferi* and *B. henselae* antigens at titers of 32. The authors speculated that co-infection may be tick transmitted; however, contact with other arthropod species should be considered. Although the detection of *B. henselae* DNA in the CSF of these patients could be attributed to amplification of DNA from nonviable organisms or to laboratory error, the repeated documentation of *B. henselae* in blood and in CSF of a young woman with a previous diagnosis of classical cat-scratch disease support the potential that this bacterium can cause chronic intravascular and central nervous system infections in immunocompetent persons (*9*).

In a study performed in Slovenia, 86 febrile children were screened for serologic evidence of exposure to multiple tick-borne organisms within 6 weeks of a known tick bite (*35*). Acute- and convalescent-phase serum samples were collected from each child. Prior exposure was determined for 5 children who harbored *B. henselae* IgG and for 4 children who harbored *B. quintana* IgG. Seroconversion of IgG to both antigens was detected for only 1 child (*35*). Morozova et al. tested for *Bartonella* DNA in persons from the Novosibirsk region of Russia who had been bitten by ticks during the summers of 2003 and 2004 (*38*). *Bartonella* DNA closely related to *B. henselae* and *B. quintana* was detected in the blood of some patients by using *gro*EL-specific primers (*36*). A more recent study, performed by Breitschwerdt et al., screened 42 immunocompetent patients, who had had prior animal and arthropod contact, for *Bartonella* spp. (*37*) The study included 12 women and 2 men who reported having had occupational animal contact for >10 years, including frequent animal bites, animal scratches, and arthropod exposure (e.g., fleas, ticks, biting flies, mosquitoes, lice, mites, chiggers). *B. henselae* or *B. vinsonii* subsp. *berkhoffii* were detected by PCR or were cultured from all patients (*37*). Case studies and surveys of this type suggest that ticks may serve as competent vectors of *Bartonella* spp., but this supposition cannot be confirmed until experimental studies demonstrating successful transmission have been performed.

Recently, Cotté et al. detailed the potential transmission of *B. henselae* by *I. ricinus* ticks (*38*). Using an artificial feeding platform made of rabbit skin, the authors successfully (based on PCR screening) infected ticks with *B. henselae* of molted ticks previously fed infected blood, suggesting that transstadial transmission may be possible. Subsequently, molted ticks were placed onto rabbit skins and fed noninfected blood, after which *B. henselae* was either cultured or detected by PCR analysis within 72 hours of when aliquots were taken from the previously noninfected blood. This finding indicates that during a blood meal, the organism could potentially be transferred from an infected tick to a noninfected individual. In addition, *B. henselae* bacteria were also present within molted ticks in sufficient numbers to cause bacteremia when tick salivary gland extracts were inoculated intravenously into domestic cats. Because ticks were not allowed to attach directly to the cats, this study supports, but does not prove, tick transmission of *B. henselae* by *I. ricinus*. Consistent with the transmission of *Bartonella* spp. by other arthropods such as fleas and lice, *B. henselae* does not seem to be transovarially transmitted in ticks because larvae hatched from *B. henselae*–positive (by PCR) egg clutches did not harbor detectable *Bartonella* DNA (*2**,**38*).

## Conclusions

The number of zoonotic *Bartonella* spp. identified in the past 15 years has increased considerably. This review indicates that a diversity of *Bartonella* spp. DNA can be amplified from various tick species from numerous geographic locations, that tick attachment has preceded the onset of illness in a small number of patients from whom *B. henselae* DNA has been amplified, and that serologic and molecular evidence suggests cosegregation of *Bartonella* spp. with known tick-borne pathogens. Therefore, ticks might serve as potential *Bartonella* vectors. However, there is little evidence that *Bartonella* spp. can replicate within ticks and no definitive evidence of transmission by a tick to a vertebrate host. Only Kruszewska and Tylewska-Wiezbanowska reported successful isolation of *Bartonella* sp. from a tick (*25*); all other studies were based on amplification of *Bartonella* DNA from ticks by using PCR. As the medical relevance of the genus *Bartonella* continues to evolve, it is clearly necessary to determine whether ticks or other arthropods play a role in the transmission of *Bartonella* spp. among animals and humans. For this reason, experimental transmission studies, using infected ticks placed on live animals, are required to determine whether ticks are vector competent for the transmission of *Bartonella* spp.

## Addendum

Since the submission of this manuscript, we found 3 cases of *B. henselae* infection transmitted by *Dermancentor* spp. ticks. These patients had scalp eschar and neck lymphadenopathy (*39*).
